# New Trends for the Evaluation of Heat Treatments of Milk

**DOI:** 10.1155/2017/1864832

**Published:** 2017-11-02

**Authors:** Mena Ritota, Maria Gabriella Di Costanzo, Maria Mattera, Pamela Manzi

**Affiliations:** Consiglio per la ricerca in agricoltura e l'analisi dell'economia agraria (CREA), Centro di ricerca Alimenti e Nutrizione, Via Ardeatina 546, 00178 Rome, Italy

## Abstract

Milk is generally very rich in nutrients and this may lead it to be an ideal growth environment for many microorganisms, including pathogens, so effective measurements aiming to ensure total microbiological safety of milk and minimize the risk to human health are needed. Milk heat treatments are the most common practices carried out to inhibit the microbial growth; therefore it is necessary to have analytical procedures that are more and more up-to-date and capable of detecting the effectiveness of the heat treatments. Most of the reference and official methods to assess heat treatment in milk are based on the evaluation of the modifications of some milk components following the thermal process, such as the determination of enzyme activities (alkaline phosphatase and lactoperoxidase), whey proteins, Maillard reaction compounds (generally furosine), and lactulose. Besides the most common techniques (liquid and gas chromatography, capillary electrophoresis, or spectroscopy) used for the detection of single thermal indicators, new approaches, such as chemometric studies or more recent techniques, including size-exclusion chromatography with online electrospray mass spectrometry or stable isotope ratio mass spectrometry, are discussed in this review in order to evaluate heat treatment in milk.

## 1. Introduction

Milk is a food item rich in nutrients; it is source of essential amino acids and has significant amounts of vitamins and minerals, particularly calcium. Due to this high nutrient content and its low acidity, milk is an ideal growth environment for many microorganisms, resulting in a perishable and very easily spoiling commodity.

Heat treatments have become the most important part of milk processing, representing the common practice to inhibit the microbial growth in milk, and a classification type of milk is really based on the heat treatment to which milk is processed to.

According to the European regulation in force [1] raw milk or dairy products undergoing heat treatment have to satisfy the requirements of the hygiene of foodstuffs [2]. The same regulation [1] also specifies the temperature and time conditions to obtain correct pasteurization and UHT treatment. Pasteurization must be carried out by (i) a high temperature for a short time (at least 72°C for 15 seconds); (ii) a low temperature for a long time (at least 63°C for 30 minutes); or (iii) any other combination of time-temperature conditions to obtain an equivalent effect, such that foods show negative reaction to alkaline phosphatase immediately after the heat treatment. Ultra High Temperature (UHT) treatment must be carried out: (i) involving a continuous flow of heat at a high temperature for a short time (not less than 135°C in combination with a suitable holding time), so that no viable microorganisms or spores are capable of growing in the UHT food when it is stored in an aseptic closed container at room temperature and (ii) in order to ensure a microbiological stability of the food after incubating for 15 days at 30°C in closed containers or for 7 days at 55°C in closed containers or after any other method demonstrating that the heat treatment has been successful.

Heat treatments applied to milk have the dual purpose of making the product more healthy and extending its shelf life. However, some modification in milk may unavoidably occur following thermal process, but the heat-treated milk is undoubtedly the best compromise between safety (a basic requirement) and the chemical-nutritional and organoleptic quality of milk.

The analytical methods developed for assessing the heat load are generally based on the evaluation of the modifications in the physicochemical state of the milk components following heat treatment. Therefore, starting from the well known reference and official methods for evaluating heat treatments indicators, this review aims to provide an overview of new analytical approaches available in the literature for determining these molecules, or other single molecules with the potentiality of being considered heat treatments indicators. Among the new analytical techniques employed for assessing heat treatments in milk, particular attention is given to spectroscopic techniques (UV-Vis and fluorescence), because they are fast and do not require large sample preparation, and other analytical methodologies, principally employing mass spectrometry.

**Figure 1:**
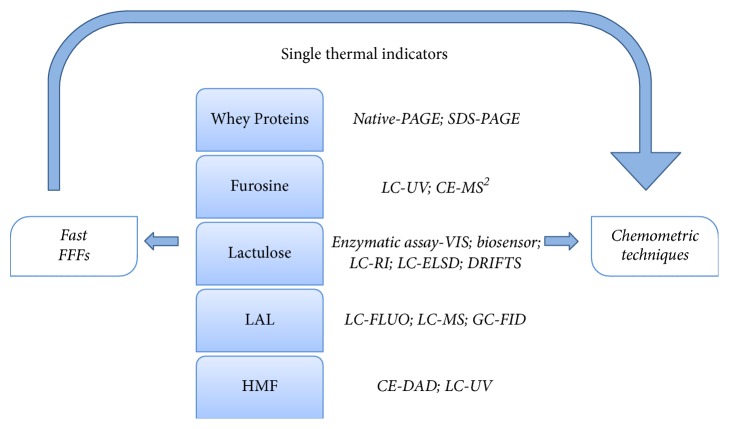
Different approaches for the evaluation of thermal treatment in milk and dairy products.

Moreover, in recent years the use of multiparametric approach has become widespread: this takes into account multiple indicators at the same time and makes use of the multivariate analysis, resulting in a more efficient description of the heat-induced changes in milk. In Figure 1 a summary of different procedures for the evaluation of thermal treatment in milk and dairy products is shown.

## 2. Reference Methods

Heat treatments cause different modifications in the chemical and sensorial characteristics of milk. The major variations primarily involved certain protein fractions (enzymes, whey proteins) and the formation of the Maillard reaction products. Therefore, the official methods for evaluating the heat treatments of milk concern the determination of these compounds, together with the evaluation of the lactose-derived compounds.

### 2.1. Proteins

#### 2.1.1. Alkaline Phosphatase

Alkaline phosphatase is a naturally occurring enzyme in raw milk that is inactivated during pasteurization (heat treatment of 72°C for 15 s, or any other temperature–time combination producing an equivalent effect). For this reason, the determination of this enzyme in milk is considered an index of the effectiveness of pasteurization.

When determining alkaline phosphatase activity for raw milk and heat-treated milk, the ISO standard [3] must be applied as reference method. This method “*specifies a fluorimetric method for the determination of alkaline phosphatase (ALP, EC 3.1.3.1) activity in raw and heat-treated whole milk, semi-skimmed milk, skimmed milk and flavoured milks. This method is applicable to milk and milk-based drinks from cows, sheep and goats. It is also applicable to milk powder after reconstitution.*” The alkaline phosphatase activity is expressed as mU/L and a unit of alkaline phosphatase activity is the amount of alkaline phosphatase enzyme that catalyses the transformation of 1 micromole of substrate per minute [4]. The reference method allows the determination of the alkaline phosphatase activity up to 7000 mU/L; otherwise the sample needs to be diluted. An alkaline phosphatase test is considered giving negative result if the measured activity in cow's milk is not higher than 350 mU/L [4].

#### 2.1.2. Lactoperoxidase

Lactoperoxidase is another milk native enzyme which is more heat-stable than alkaline phosphatase. It is inactivated at 75–80°C; therefore, if the milk has been exposed to a higher temperature (>75°C) during pasteurization, the inactivation of the enzyme will occur and a consequent negative peroxidase test will result. The method for the determination of the lactoperoxidase activity is based on the oxidation of hydrogen peroxide by peroxidase: the developed oxygen oxidizes the colourless 1,4-phenylenediamine into the purple indophenol (Storch test), with a colour intensity proportional to the enzyme's concentration [5]. If the milk is properly pasteurized a blue colour will occur within 30 seconds after mixing; otherwise no colour will occur, which means that lactoperoxidase has been inactivated following a temperature higher than that of pasteurization. The ISO method [6] specifies a photometric method for the determination of the lactoperoxidase activity in milk in amounts exceeding 50 U/L.

#### 2.1.3. Whey Proteins

Whey proteins are the most heat-sensitive among the milk constituents. They tend to denaturation and to form complexes with casein, even at relatively low temperatures. For this reason, the soluble whey proteins fraction is inversely proportional to the strength of the heat treatment. Their dosage is made, by difference, between non-caseinic nitrogen (NCN) and non-protein nitrogen (NPN) expressed as total nitrogen, both NCN and NPN being carried out according to the Kjeldahl method, that is, the reference method for the determination of the nitrogen content in milk and milk products [7].

### 2.2. Maillard Reaction Products

The Maillard reaction, the main nonenzymatic browning, which involved a chemical reaction between an amino group and a reducing sugar, usually requires the addition of heat. In milk, the Maillard reaction occurs between the free aldehyde group of the glucose unit in the lactose molecule and the amine group of lysine residues, generating *ε*-lactulose-lysine (Amadori product) as the first stable product [8]. This compound is analytically determined by conversion into furosine by hot acid hydrolysis using a HPLC method developed by Resmini et al. [9] and subsequently published as an ISO standard.

#### 2.2.1. Furosine

It is commonly used as an indicator for assessing the effects of the thermal treatments applied to milk. Its content is determined by ion-pair reverse-phase high performance liquid chromatography method (RP-HPLC), according to the ISO standard method [10], with UV detector at 280 nm. This method is particularly applicable to raw or heat-treated milk and to cheese.

The greater the heat treatment applied to milk, the higher the furosine content that can be analytically determined and the lower the bioavailability of lysine in milk.

### 2.3. Lactulose

Lactulose, a molecule not present in raw milk, is formed by epimerization of lactose due to heat treatment. The isomerization process is closely linked to the pH, time, and temperature of the thermal treatment; therefore the lactulose determination is intended to evaluate the severity of the heat treatment of milk.

Lactulose content can be determined according to an enzymatic [11] or an HPLC ISO method [12]. Regarding the enzymatic method, the sugars present in milk are hydrolysed by the *β*-D-galactosidase, to determine the amount of potentially released fructose. The enzymatic methods allow measuring numerous samples simultaneously; however other carbohydrates such as fructose and lactose may interfere resulting in an overestimate of lactulose [13, 14]. The HPLC ISO method [12] is to be used in cases of dispute and is specific for the determination of the lactulose content of heated milk, skimmed, partially skimmed, or whole milk, in order to distinguish milk sterilized by ultra-heat treatment (UHT) from in-bottle sterilized milk. Moreover, the HPLC methods allow the simultaneous determination of both lactulose and other sugars without interferences of other carbohydrates [13].

## 3. Nonreference Methods Based on Single Thermal Treatment Indicators

### 3.1. Whey Proteins

A work of Lin et al. [15] evaluated whether native-PAGE (polyacrylamide gel electrophoresis) was suitable for studying the whey proteins of several milks processed at different temperatures. The whey proteins, obtained after precipitation by lactic acid (pH 4.6) and casein removal by centrifugation, were evaluated by both native-PAGE and SDS-PAGE (polyacrylamide gel electrophoresis in the presence of sodium dodecyl sulphate).

In this work [15] SDS-PAGE was employed to evaluate the total milk proteins (both caseins and whey proteins); however, according to the authors whey protein bands did not clearly get recognized in SDS-PAGE. Native-PAGE, instead, allowing the maintenance of the protein native structure, identified the* real* protein, providing a useful tool to detect changes due to heat treatments.

The results showed that the amount of whey proteins decreased during heat treatments (less than 23% in pasteurized milk to over 85% in UHT, compared to the raw milk). *α*-lactalbumin (*α*-LA) was less sensitive to heat (32% is in its native state after heating at 100°C for 10 min). Approximately 42% of *β*-lactoglobulin (*β*-LG) variant A and 53% of variant B were denatured at 75°C for 30 min, while blood serum albumin (BSA) was almost completely denatured after 30 min when milk at pH 5.0 was heated to 75°C or higher temperatures. The authors concluded that native-PAGE, a convenient and low cost technique, could be suitable for the routine separation and quantification of the whey proteins both in raw and heat-treated milk, allowing the identification of the changes according to the temperature, time, and pH of the treatment.

### 3.2. Furosine

Bignardi et al. [16] developed a method for the qualitative and quantitative analysis of furosine in food products using capillary electrophoresis (CE) coupled to Tandem Mass Spectrometry (CE-MS^2^). Unlike most works in literature concerning furosine analysis by CE, the authors did not use any sample SPE pretreatment. Milk samples were hydrolysed with HCl 8 N and then resuspended in the running buffer (formic acid 50 mM, pH 2.7), in order to obtain a pH value of the sample similar to that of the mobile phase, thus avoiding any interactions of the analyte with the silanol groups of the inner wall of the fused silica capillary. Separation was performed using two different untreated fused silica capillaries (50 *μ*m id) with effective length of 90 and 60 cm, pretreated before use and washed after each run. UV detection was performed at 280 nm and quantitative analysis was carried out by means of a calibration curve built in the matrix using the internal standard method. The authors compared the results obtained with the CE-MS^2^ method with those obtained with the reference method (by means of HPLC), showing both time and cost advantages. Moreover, the method performances were satisfactory: LOD and LOQ values were equal to 0.07 and 0.25 mg/L, respectively; recovery was 77% at 4 ppm and 97% at 16 ppm; intra- and interday repeatability (RSD%) were equal to 4–6% and 14–16%, respectively. The authors concluded that the developed electrophoretic method could be a valid technique for fast, reliable, and low cost analysis of furosine content in food samples.

### 3.3. Lactulose

Different analytical techniques are available in the literature to detect lactulose. The earliest were based on the enzymatic determination with spectrophotometric detection. Marconi et al. [17] developed a simple, rapid, and sensitive method for determining lactulose in milk based on a spectrophotometric-enzymatic assay after treatment with Carrez I and II reagents. Lactulose was hydrolysed into fructose and galactose by *β*-galactosidase, and fructose, in the presence of fructose dehydrogenase and a tetrazole salt (MTT), formed a colourable compound detected at 570 nm. The authors obtained a detection limit of 2.5 mg/L for lactulose, suggesting that the developed method could be readily adapted to an enzymatic test kit.

In a previous work, Moscone et al. [18] developed a simple and rapid flow system, where *β*-galactosidase was immobilized in a reactor and the amount of the released fructose was measured using an electrochemical biosensor based on the fructose dehydrogenase enzyme. The method had a limit of detection of 5 × 10^−7 ^mol/L for fructose in milk and allowed the direct measurement of lactulose in the range 1 × 10^−5^–5 × 10^−3 ^mol/L with no milk pretreatment. The method was also able to discriminate the different heat-treated milks (pasteurized, UHT, and in-container sterilized).

Later, Luzzana et al. [19] proposed a very simple and fast method for the determination of lactose and lactulose in milk based on the measurement of the pH variations following enzymatic reactions by *β*-galactosidase, glucokinase, and hexokinase. The observed pH variations were found to be proportional to the lactose and lactulose content of milk. The method had a limit of detection of 0.1 mmol/L for lactulose and allowed in less than two and four minutes, respectively, the lactose and lactulose determination with no sample pretreatment.

In most cases, the determination of lactulose is based on chromatographic techniques. The main problems concerning the determination of lactulose in milk with this approach are the presence of a lactose amount two orders of magnitude larger than lactulose content and a similar retention time of the two compounds. The HPLC method proposed by Manzi and Pizzoferrato [20] allowed a good separation between lactose and lactulose by using two in-series amino-based columns (150 × 4.6 mm, 3 *μ*m) at 35°C, an isocratic elution with acetonitrile/water (75 : 25, v : v) at a flow rate of 1.0 mL/min and a detection with refractive index at 35°C. Milk samples were pretreated with Carrez I and II reagents prior to injection. The method allowed a determination of the milk lactulose content over a linear range of 0.060–1.006 mg/mL and showed LOD and LOQ of 0.013 mg/mL and 0.028 mg/mL, respectively.

Schuster-Wolff-Bühring et al. [21], instead, developed an HPLC method for lactose and lactulose evaluation in milk by Evaporative Light Scattering Detection (ELSD). A HPLC column with an amino-bonded polymeric matrix (250 × 4.6 mm) yielded good results using a mobile phase of acetonitrile/water (70 : 30, v : v) at a flow rate of 0.9 mL/min at 25°C. The method allowed obtaining a lactose and lactulose detection limit of 3.8 and 2.5 mg/L, respectively, and was successfully applied on commercial milk samples.

The same column was recently used by Silveira et al. [22] in order to develop a method for the simultaneous determination and quantification of lactulose and lactose in commercial UHT and sweetened condensed milk. The chromatographic conditions slightly differed from the study of Schuster-Wolff-Bühring et al. [21]: the mobile phase was a mixture of acetonitrile/water (75 : 25, v : v) at a flow rate of 1.1 mL/min; column was kept at 30°C with a refraction index detector.

Recently, Pappas et al. [23] determined lactulose content in freeze-dried heat-treated milks using Diffuse Reflectance Infrared Fourier Transform Spectroscopy (DRIFTS), which has the advantage of not requiring any sample pretreatment. The spectral region 1286–754 cm^−1^ of DRIFTS spectra in the second-derivative form was correlated with the lactulose values determined by HPLC: using Partial Least Squares (PLS) regression, the authors established a linear correlation (*R*^2^ = 0.997) between the HPLC values of lactulose and the concentrations recalculated through the PLS model: having the appropriate instrumentation available, this method [23] is simple, fast, and low cost.

### 3.4. Lysinoalanine

Heat treatment can also induce interactions between proteins, resulting in the formation of new compounds which were not present in milk. Lysinoalanine (LAL) is generated by the spontaneous lysine condensation with dehydroalanine, which is in turn generated by *β*-elimination of the residues of phosphoserine, with release of phosphate, or cysteine with release of H_2_S. The reaction is generally favoured in an alkaline medium, but by heating the casein *α*s1 at 120°C for 30 minutes LAL formation is also observed in a neutral environment [24].

It is well known that LAL reacts with dansyl chloride to form a fluorescent product which can be determined by liquid chromatography with fluorimetric detector [25]. Recently this method was also employed by Karami et al. [26] to determine LAL in infant formula. The authors obtained a limit of detection equal to 2 mg/L, with a recovery ranging between 83.6 and 87.7%.

Values higher than 50 ppm of LAL have been associated with the presence of caseinates in cheeses. According to Pellegrino et al. [27], LAL was not present in pasteurized milk but values between 0.4 and 4 ppm were found in mozzarella cheese, by analysis with HPLC and fluorimetric detection after derivatization with 9-fluorenyl-methylchloro-formate (FMOC). Furthermore, the authors highlighted the higher content of LAL in different types of processed cheeses and* “imitation”* mozzarella cheese (from 15 to 421 ppm).

Calabrese et al. [28] proposed an advance of the method of Pellegrino et al. [27], developing a HPLC-ESI/MS method for determining lysinoalanine with the selective ion monitoring of the FMOC-LAL derivatives, after acid hydrolysis. Compared to HPLC, MS has the advantage of avoiding most false positives due to the interference of coeluting compounds, thanks to the accurate molecular weight measurements. The method was applied to different dairy products (natural and adulterated milk and pasta filata cheeses), showing that LAL was not present in raw milk or derived from mozzarella cheese, while high amounts of LAL were found in calcium caseinate and milk powder.

An alternative method for determining LAL was proposed by Montilla et al. [29]. In order to simplify the pretreatment of the sample before the analysis, the authors developed a GC-FID method for the analysis of LAL in different food items (boiled eggs, commercial caseinates, fresh cheese, fresh cheese made from milk supplemented with caseinate, and fresh cheeses adulterated with caseinate after cheese making). In their method an acid hydrolysis of the sample was carried out and followed by derivatization; according to their results, LOD and LOQ were, respectively, 50 and 152 ppm of LAL/protein.

### 3.5. 5-Hydroxymethylfurfural

5-Hydroxymethylfurfural (HMF) is an intermediate of the Maillard reaction, and it is widely used as an indicator for assessing heat treatment in dairy products. HMF is mainly formed by acid degradation of sugars where Amadori products degradation is a minor route [30]. When talking about HMF, a distinction has to be done between free HMF and total HMF. Total HMF is the sum of the HMF precursors (lactulosyl-lysine, 1-2 enolized products, etc.) and free HMF [30]; its determination requires a preliminary digestion with 0.3 N oxalic acid at about 100°C to convert the Amadori products into HMF. The acidic digestion generates HMF mainly from the Amadori compounds but also from other Maillard reaction intermediates [31]. For the determination of free HMF the hydrolysis step is omitted [30].

A rapid and cost-effective method for HMF determination involved the reaction with thiobarbituric acid (TBA) [32]. However, this colorimetric method is not specific for HMF: TBA reacts with aldehydes and not only HMF is formed during the processing of milk [33]. Furthermore, the reaction product is unstable; therefore a strict control of temperature and time reaction is required [34]. To avoid instability of the results, HPLC technique is widely employed: it is more accurate, it does not require a derivatization step, since HMF has a strong absorption at about 280 nm, and it allows the simultaneous determination of all furfural compounds [34].

Morales et al. [35] proposed an HPLC method for total HMF determination in industrial processed milk: samples were digested with oxalic acid (0.3 N) for 1 h at 100°C, then were deproteinized with trichloroacetic acid solution (40%, w/v), and filtered prior to the HPLC determination with detection at 280 nm. The detection limit of the method was 0.2 *μ*mol/L.

Chen and Yan [36] proposed a potentially rapid and reliable method to simultaneously determine both melamine and HMF in milk by capillary electrophoresis with diode array detection (214 nm for melamine and 280 nm for HMF). In order to improve separation efficiency, sodium dodecyl sulphate (SDS) was introduced into the electrolyte and micellar electrokinetic capillary chromatography was employed for enhancing resolution. Milk samples were extracted with 1% trichloroacetic acid and then centrifuged and filtrated prior to the CE-DAD analysis. The proposed method was suitable for melamine and HMF determination in a wide linear dynamic range (0.05–100 *μ*g/mL and 0.1–100 *μ*g/mL, resp.), and the calculated detection limits were 0.047 *μ*g/mL for melamine and 0.067 *μ*g/mL for HMF.

Gökmen and Şenyuva [37] developed a LC-MS method with positive atmospheric pressure chemical ionization (APCI) for the determination of HMF in baby foods. Sample preparation involved aqueous extraction from food with simultaneous clarification using Carrez I and II reagents and solid-phase extraction clean up using Oasis HLB. Sample preparation and analytical determination were completed in <20 min, while recovery ranged between 91.8 and 94.7%.

### 3.6. Free Thiol

In the detection of an adulteration of raw milk samples by milk powder or reconstituted milk, hydroxymethylfurfural is the index used routinely as a reference [38]. However, Muangthai and Surapat [39] proposed an alternative approach based on the determination of the free thiol content in the whey proteins isolated from heat-treated milk. *β*–LG is the main whey protein; it is composed of 162 amino acid residues, with two disulphide bonds (Cys 66–Cys 160 and Cys 106–Cys 119) and a free thiol group at Cys 121. This free thiol is masked by the C-terminal of a segment of *α*-helix in the native protein [40], but it changes following heat treatment of milk, due to the interactions between the reactive free thiol groups which result in the formation of polymers or new substances [41]. Free thiol groups can be easily determined by spectrophotometric techniques, since they rapidly react with 5,5′-dithiobis (2-nitrobenzoic acid, DTNB) under alkaline conditions to form a stable yellow product [42].

In their work Muangthai and Surapat [39] evaluated the possibility of using the free thiol content as a potential indicator for assessing heat treatment in milk. The authors determined the free thiol contents of raw milk, reconstituted milk, and mixed milk samples after pasteurization (LTLT 60–65°C × 30 min; HTST 82–85°C × 2–5 sec) and UHT processes. Caseins were precipitated with acetic acid, incubated at 45°C for 15 min, and mixed with sodium acetate. Caseins and denatured whey proteins were removed by filtration and the filtrate, containing undenatured whey proteins, was analysed for the free thiol content. The absorbance of the filtrate was measured after the addition of DTNB 5,5′-dithiobis-(2-nitrobenzoic acid) solution in ethanol against a blank at 412 nm. The authors showed that the free thiol content was able to discriminate between raw milk and reconstituted milk, both after heat treatments. In particular, in reconstituted milk the thiol content was significantly lower, and the results showed a decrease in thiols with increasing temperature in all heat-treated samples. According to the obtained results, the authors proposed this method as a useful tool to identify the adulteration of milk by reconstituted milk.

The same milk samples were also analysed for the HMF content, after deproteinization with TCA and oxalic acid, by means of HPLC. The free thiol content showed a high correlation with the free HMF content (*R*^2^ = 0.98); therefore the authors proposed the method for the determination of free thiols as a valid alternative to the HMF content determination method, since it appears to be much faster and less expensive.

A summary of the LOD and LOQ, obtained with nonreferenced methods, for the determination of some indicators of heat treatment in milk and dairy products is reported in Table 1.

## 4. Nonreference Methods Based on Multiple Heat Treatment Indicators Combined with Chemometrics

Although different methods have been reported in the literature to identify heat treatments in milk, no unique method has yet been identified. Thermal indicators alone are not often able to give an efficient description of the heat-induced changes in milk, and many of them are only suitable for assessing the severity of heat treatment soon after processing due to their changes during storage. Storage conditions play an important rule on the product quality (e.g., storage can induce gelatinization, fat separation, or sedimentation in UHT milk) and high storage temperatures are able to reduce the shelf life of milk [43].

Le et al. [44] reported that heat treatment indicators can change during storage: milk protein concentrate powder (containing 80% protein), stored at temperatures ranged from 25 to 40°C and relative humidity ranged from 44 to 84%, showed a decrease in furosine and free HMF. Moreover also lactulose content was affected by the storage temperature: the increase of lactulose was about 1.5% in UHT milk stored at 20°C for 8 weeks and about 7% if stored at 32°C [45].

For this reason, many authors tried to apply chemometric approach in order to have an overview of the system and more efficiently describe the effects of heat treatment in milk.

In this context, Morales et al. [35] studied the effects of different heat treatments on milk by simultaneously evaluating several thermal damage indices: HMF, lactulose, and acid-soluble whey proteins (*β*-LG, BSA, and *α*-LA). The same bulk milk was subjected to different industrial heat treatments (thermization, pasteurization at 85°C for 30 s, direct and indirect UHT sterilization, presterilization, and in-bottle sterilization). Whey proteins were determined after caseins precipitation at pH 4.6 and dilution with phosphate buffer by HPLC, using a PLRP-S column (150 × 4.6, 8 *μ*m) at 40°C and a gradient elution (solvent A: trifluoroacetic acid (0.1%) in water, solvent B: trifluoroacetic acid (0.1%) in acetonitrile) at 1 mL/min, with detection at 205 nm. Lactulose was determined after protein precipitation by glacial acetic acid and ethanol by means of HPLC, using a Carbohydrate column (3.9 × 300 mm, 10 *μ*m) at 30°C with a mobile phase of acetonitrile/water (80 : 20, v : v) at 2 mL/min and a refractive index detector (30°C). HMF was finally determined by HPLC after hydrolysis by oxalic acid at 100°C for 3 h and deproteinization with TCA (40%); separation was performed on an ODS-2 column (250 × 4.0, 5 *μ*m) with a mobile phase of sodium acetate (0.08 M pH 3.6) and a detector set at 280 nm. In this work Morales et al. [35] observed that under severe heat treatment conditions the whey proteins content rapidly decreased, up to 100% denaturation during the in-bottle sterilization process. Lactulose, absent in raw milk and below the quantification limit in pasteurized milk, was present in UHT milk (higher in indirect than direct heating) and even more in the in-bottle sterilized milk. A low content of HMF was observed in pasteurized (at 85°C for 30 s) and thermized milk. Although the three thermal damage indicators provided a useful tool of measuring heat-induced changes in milk, the authors are aware that these indices alone have some limitations: when measuring thermal damage indicators, in fact, pasteurized milk (at 85°C for 30 s) and UHT (direct and indirect) are grouped together due to a not significant difference in the observed results. Resorting to a multivariate statistical approach allowed for an overview in describing the analytical system: when applying Discriminant Analysis (DA), in fact, pasteurized, UHT, presterilized, and in-bottle sterilized milks were clearly separated, with 100% accuracy. The main conclusions of this study showed that heat treatment damage occurring in milk can be evaluated through several closely related indexes: in particular, *β*-LG is a good index for mild treatments, which resulted in the formation of low amounts of lactulose and HMF. These two indicators are instead more effective for assessing severe heat treatments. Discriminant Analysis allowed classifying all the heat-treated milks with a confidence level of 95%, except for UHT milk, in which direct and indirect heating are not discriminated.

In the work of Feinberg et al. [46], a statistical analysis was applied to several selected indicators of heat treatments, including furosine, lactulose, native *α*-LA, denaturised *α*-LA, FAST index, tryptophan fluorescence (F_Trp_), *β*-LG, and lactoperoxidase, determined in 200 commercial samples of heat-treated milks (pasteurized, high pasteurized, direct UHT, indirect UHT, and sterilized milk) and subjected to storage. The authors collected 5000 measurements and a 4-factor nested experimental design, technology (5 levels), season (2 levels), time of the process (2 levels), and storage (3 levels), was applied. Analysis of variance (ANOVA) allowed the identification of the statistically significant differences between the studied indexes, but it showed that no tracer could be selected to universally discriminate all the heat-treated milks. Discriminant Analysis was helpful in the authentication of the heat treatments in milk. The authors found that all milk samples could be properly classified by combining two indicators (F_Trp_ and *α*-LA), but only in not stored samples, since storage conditions, as it is well known, modify the concentrations of the different indicators and changed the reference values. The use of at least five indicators, instead, allowed a good discrimination: this reflected the sensitivity of the Discriminant Analysis to the number of the data used to build the model. The results also showed that the indicators with a higher discriminating power were the ones that measured the structural changes in milk proteins, compared to those measuring the Maillard reaction's compounds.

## 5. Nonreference Methods Based on Spectroscopic Techniques

In recent years, spectroscopic techniques have been widely used in the food quality control, especially because they are fast and do not require large sample preparation. In dairy science they have also been employed for measuring the effects of heat on milk.

### 5.1. UV/Vis Spectroscopy

Heat treatment causes immediate UV absorbance of amino-sugars mixtures and absorbance at 294 nm is often used to determine the intermediate compounds of the advanced Maillard reaction [47]. Therefore, the determination of the UV absorption of model systems (heat-treated amino-sugar mixtures) could be an alternative and rapid method to evaluate the heat treatment intensity. Sun and Wang [48] proposed an UV-Vis method to evaluate the effects of thermal processes in milk and milk-like systems by measurements of the UV absorbance of amino-sugars mixtures processed at different temperatures (100, 120, and 140°C) and holding times (from 0 to 30 minutes), after hydrolysis. The method was validated by correlating the absorbance values of these milk-like systems with the furosine content determined by HPLC method [9]. The authors found good correlations between the two values in samples heated at 100°C for 0–30 min (*R*^2^ = 0.9569, *P* < 0.001) and 120°C for 0–20 min (*R*^2^ = 0.9594, *P* < 0.01); furthermore they observed an increase in the maximum absorbance (*A*_max_ at 294 nm) of the heat-treated milk-like systems with increasing intensity of the heat treatment and a linear increase with time for a constant temperature. While furosine determination is inaccurate in the advanced stage of the Maillard reaction, since its content tends to decrease, the UV-Vis method showed more consistent results under severe heat treatment. Applied to commercial milks, the method allowed the discrimination between pasteurized and UHT samples in a shorter analysis time (only 3 min) compared to the furosine-based method. Correlations between the *A*_max_ and the furosine content of commercial samples were *R*^2^ = 0.8196 (*P* < 0.01) for pasteurization and *R*^2^ = 0.4586 (*P* < 0.05) for UHT sterilization, showing how this method [48] had better performances under mild heating conditions.

Spectrophotometric technique was also employed by Neves et al. [49] to monitor the whey proteins solubility and the Maillard reaction during the heat treatment in commercial UHT milk. The whey protein nitrogen (WPNI) and the 5-hydroxymethylfurfural (HMF) content in the visible region were determined and chemometric tools were used in the data analysis. The regression models built identified a Confidence Interval (CI) of 0.91–3.73 mg/mL for the WPNI, while for free HMF the CI were 2.39–3.27 and 6.01–6.89 *μ*mol/L for regular and low lactose content samples, respectively. The authors observed a lactose content influence in the total HMF index: the relevant CI for the regular and low lactose content samples for total HMF index were, in fact, 11.67–12.39 and 132.28–143.22 *μ*mol/L, respectively. Anyway, the proposed method allowed obtaining information about the heat treatment applied to UHT milk and therefore about its quality.

### 5.2. Fluorescence Spectroscopy

The observed association between fluorescence and the nonenzymatic browning has encouraged in recent years the application of the fluorescence spectroscopy in the evaluation of heat treatments applied to milk. Birlouez-Aragon et al. [50] developed a new fluorimetric FAST (fluorescence of Advanced Maillard products and Soluble Tryptophan) method to evaluate the intensity of heat treatment in milk, based on the quantification of protein denaturation by fluorescence measurements of tryptophan (F_Trp_) and accumulation of fluorescent Maillard products (F_AMP_) in the pH 4.6-soluble fraction of milk. Milk was mixed with sodium acetate buffer (0.1 M, pH 4.60) and left for 5–10 min at room temperature; on the supernatant fraction the fluorescence measurement was carried out after filtration. The tryptophan fluorescence was measured at excitation/emission 290/340 nm and fluorescence values were expressed as equivalent g/L proteins using external calibration with BSA. The fluorescence of the advanced Maillard products was instead measured at excitation/emission 330/420 nm. The percentage ratio between F_AMP_ and F_Trp_ was the FAST index. The FAST method was validated on industrially processed milk samples with different sterilization systems and various temperature and holding times by comparison with standardized techniques, such as the determination of *β*-LG, furosine, and lactulose. The FAST method proved to be very rapid (100 samples per day), of low cost, and efficient, with results comparable to the other three based-indicators methods in predicting pasteurization and sterilization effects on milk. Therefore the authors proposed the F_Trp_ and the FAST index as cheaper and faster alternatives to the classic heat treatment indicators, such as *β*-LG, furosine, or lactulose determinations.

When analysing milk with fluorescence spectroscopy, a disadvantage could be the sample pretreatment before the fluorescence measurement, in order to avoid the turbidity of the sample. This inconvenience has been overcome by a new advanced fluorescence technique, the Front-Face fluorescence spectroscopy (FFFs), in which the angle of incidence changes from 90° to 56° to reduce the scattering effect; in this technique no sample preparation before the fluorescence measurement is needed.

Formerly, in 2002, FFFs was employed for developing a rapid and nondestructive method able to measure furosine and lactulose content in heat-treated milk [51]. The method involved the measurement of the fluorescence spectra directly on the milk samples (overheated semiskimmed UHT milk, semiskimmed UHT milk, and pasteurized milks), without any sample pretreatment: emission spectra (305–450 nm) of tryptophan residues were recorded at 290 nm excitation wavelength, while emission (380–600 nm) and excitation (250–420 nm) spectra of fluorescent Maillard reaction products were recorded at excitation and emission wavelengths set at 360 nm and 440 nm, respectively. Principal Component Analysis (PCA) performed on the spectroscopic data clearly showed a samples discrimination according to the heat treatment, while Principal Component Regression (PCR) showed high correlations between the results obtained by the FFFs method and those obtained by reference methods (HPLC furosine content, *R*^2^ = 0.956; enzymatic lactulose determination, *R*^2^ = 0.987).

Later, Kulmyrzaev et al. [52] assessed the use of FFFs to evaluate the effects of mild heat treatments on milk by means of the emission and excitation spectra of the fluorescent compounds naturally occurring in milk. The native fluorophores that contributed to milk fluorescence are the aromatic amino acids (tryptophan, tyrosine, and phenylalanine) and the NADH and FADH coenzymes. Similarly to the previous work of Kulmyrzaev and Dufour [51], the fluorescence measurements were directly performed on the milk samples. Emission spectra were recorded in the ranges 280–480 nm (excitation: 250 nm) and 380–600 nm (excitation: 360 nm), while excitation spectra were recorded in the range 290–490 nm at an emission wavelength set at 518 nm. The results showed a shift in the maximum of emission wavelength of tryptophan from 342 nm in the raw milk to 343 nm in the heat-treated milk, due to the effects of thermal process, while for the aromatic amino acids the spectra overlapped in the different heat-treated milks. The fluorescence spectra of the NADH and FADH coenzymes, instead, showed differences depending on the heat treatment applied to milk. PCA was therefore applied to the normalized fluorescence spectra in order to reduce the dispersion effect and successfully discriminated the milk samples according to the temperature and the time of the heat treatment. In the same work, the authors also determined, by enzymatic and immunochemical techniques, alkaline phosphatase, lactoferrin, immunoglobulin G, BSA, *β*-LG, and *α*-LA. Therefore, Principal Component Regression analysis was applied to predict the amount of the native proteins using fluorescence data: the authors observed a strong correlation between the measured data and the predicted data for alkaline phosphatase and *β*-LG.

Front-Face fluorescence spectroscopy was also employed by Schamberger and Labuza [53] to monitor the development of the Maillard reaction compounds in milk during heat treatment in a pilot plant thermal processing system, in which raw skimmed milk was processed to different heat treatment conditions. Emission spectra of tryptophan were acquired in the range 305–450 nm at an excitation wavelength of 290 nm, while emission spectra of fluorescent Maillard compounds were recorded in the range 380–600 nm at an excitation wavelength of 360 nm. All fluorescent measurements were directly performed on the milk samples, without any sample pretreatment. Furthermore, on the same milk samples the authors determined the HMF content, by spectrophotometric measurements (*λ* = 443 nm), and the colour changes due to heat treatments, through optical density of brown pigments (*λ* = 420 nm) and Hunter *L*^*∗*^, *a*^*∗*^, and *b*^*∗*^ colour coordinates. The results showed that HMF values increased with higher time/temperature combinations and a similar trend was observed for the fluorescence levels of the brown compounds. An opposite trend, instead, was observed in the tryptophan fluorescence, which showed a decrease with increasing time/temperature: it is in fact considered an index of protein denaturation. The authors found a good correlation between the FFFs data of the brown compounds and the HMF content, where *R*^2^ was greater than 0.95 in the emission spectrum region between 394 and 447 nm. Among the different methods evaluated for distinguishing differences between the several heat-treated milks, HMF analysis and Maillard browning by FFFs resulted to be the best. Moreover, since FFFs has the advantage of not requiring sample pretreatment, the authors proposed this method as the best suitable for an online instrument for monitoring and controlling the thermal processing of milk.

In a recent work by Mungkarndee et al. [54] the fluorescence induced by fluorophore/protein interactions was evaluated for its ability to discriminate milk samples according to the heat treatment (pasteurized, sterilized, UHT, and recombined milk) and according to the different type of milk (fermented, soy, and corn milk). Fluorophore solutions (5 *μ*M) and pure protein solutions (BSA, *α*-casein, *β*-casein, *α*-LA, and *β*-LG) were prepared in sodium phosphate buffer (10 mM, pH 7.4). In order to acquire the emission fluorescence spectra between 400 and 600 nm, at a 375 nm excitation wavelength, fluorophore aliquots were added to the pure protein solutions and to the diluted milk samples. A PCA was applied to the fluorescence data in order to evaluate the ability of the fluorophores in discriminating milk samples: results showed a good separation between samples both in the case of pure proteins and in the different type of milk samples. Linear Discriminating Analysis (LDA) was performed on the same data, which, after selecting the most discriminating fluorophores, showed excellent results in the discrimination of the milk samples according to the heat treatment (100% in accuracy in cross-validation with the leave-one-out technique).

## 6. Nonreference Methods Based on Other Analytical Techniques

Maillard's reaction can induce some changes in proteins, such as conformational or chemical modifications and aggregation, which can be detected by different analytical techniques. A work of Johnson et al. [55] employed the native size-exclusion chromatography with online electrospray mass spectrometry (SEC-ESI-MS) to evaluate the effects of the heat treatments on different milks proteins. SEC is based on the absence of chemical interactions between the analyte and the stationary phase; therefore it provides an ideal solution for the separation of intact proteins. These authors [55] studied different commercial milks, including pasteurized, UHT milk, skimmed milk powder, and infant formula; fats were removed from samples, and standard of *α*-LA, *β*-LG, and *β*-casein was used for the identification of proteins. Different peaks with UV absorbance at 220 nm were detected: mass spectra deconvolution allowed the assignment of the protein composition of these peaks, including caseins, *β*-LG, and *α*-LA. An aggregate material was also detected: the relevant peak appeared to be composed of high MW material (>150 kDa) and it is likely to correspond to heavily cross-linked proteins due to the *β*-sheet and disulphide bridges interactions following heat treatments. According to the proteins SEC behaviour, the authors could classify the commercial milks into three groups: (i) raw and pasteurized milks, where the proteins were mostly in a native state due to no or minimal heat treatment; (ii) moderately modified thermally processed liquid milks, in which a lower level of *α*-LA and *β*-LG was observed compared to the previous group; (iii) extensively modified milks, comprising milk powders, in which *α*-LA and *β*-LG were still resolved in separate peaks but were present in lower amounts than the other milk samples. In all milk samples caseins remained in their native oligomeric state, with the exception of an extensively thermally modified infant formula milk. The authors proposed the method as a quick tool for providing a “fingerprint” related to the heat treatments applied to milk and as a potential quality control technique in the food industry. However, it is worthwhile mentioning that this piece of equipment is very expensive and the technique requires highly qualified personnel, so it could not be suitable for small food processing companies.

A different analytical approach was proposed by Scampicchio et al. [56], who simultaneously identified milks according to the heat treatment and the geographical origin by means of stable isotope ratio mass spectrometry (IRSM). The isotope ratios of carbon and nitrogen (*δ*^13^C and *δ*^15^N) are of particular interest. Since *δ*^13^C value in milk is depending on the animal feed while *δ*^15^N value is influenced by factors such as soil conditions and the use of fertilizers, it is reasonable to expect a correlation between stable isotopes of milk and its geographical origin. The correlation between the isotopic ratios in milk or its fractions (casein, fat, and whey) with the thermal treatments is instead less known: therefore the authors [56] investigated the possibility that physical changes, occurring during heat treatments, may induce modifications in the stable isotope ratio in the individual milk fractions. *δ*^13^C and *δ*^15^N were evaluated both in milks, processed to different heat treatments (raw milk, pasteurized-HTST, and UHT) and coming from different Italian provinces (Bolzano, Udine, and Mantova), and in their fractions (fat, casein, and whey). ANOVA on the isotopic data showed differences between raw and processed milks, but it was unable to identify differences between the processed samples (HTST versus UHT); therefore the authors resorted to multivariate statistical analysis techniques. Principal Component Analysis showed that samples were clearly grouped according to the heat treatments and the geographical origin, even when simultaneously considering the effects of the process and origin. Linear Discriminant Analysis was employed to build a classification model on the samples from Bolzano and on a reduced number of initial variables (*δ*^15^N of whey and fat and *δ*^13^C of casein), in order to minimize overfitting: it resulted in 100% of successfully recognition in cross-validation. Multiple Linear Regression (MLR), Principal Component Regression (PCR), and Partial Least Squares (PLS) were applied to the full data set in order to build regression models. The predictive capability of the MLR and PLS models was better than the PCR model. Furthermore, *δ*^15^N values of the whey and fat fractions were the most important predictor for heat treatments identification, while *δ*^15^N of the whey and *δ*^13^C of the casein fractions proved to be the most effective in predicting the geographical origin of milk.

## 7. Additional Topics Concerning the Heat Treatments: Food for Thought

The EU legislation in force regarding UHT milk is deficient: for example, any changes in quality parameters (chemical, physical, and sensorial properties) of sterilized milk are not defined as well as it is lucking in the upper limit of the temperature applied to milk, the storage conditions, or the holding time of heating. The use of the appropriate time/temperature binomial in milk heat treatment is important not only for the microbiological safety but also for ensuring a quality product with high sensorial and nutritional characteristics [57] and for this reason the technologies are still developing.

Due to the improper use of time/temperature binomial, the overprocessing of milk may often occur. Based on this issue, some authors [58] studied different UHT milks processed to direct and indirect heating, also evaluating the effects of milk recirculation as well as changes occurring in commercial UHT milks during storage (up to 90 days). The authors evaluated the furosine content [10], the lactulose content [12], the galactosyl-*β*-pyranone (GAP) content [59], and the lysinoalanine content [27] in all milk samples. The results showed that all the indicators increased, when severe heat treatments, such as UHT, applied. Lactulose and furosine levels were found to be consistent with UHT milk values, but in the case of overheating or high percentage (60%) of milk recirculation GAP considerably increased (due to Maillard's advanced reaction). Therefore, the authors identified galactosyl-*β*-pyranone as an effective parameter to identify milk overheating. Lysinoalanine, instead, proved to be the most sensitive parameter to storage conditions.

In a work of Lan et al. [60], the effect of the different temperatures and the use of reconstituted milk on different heat treatment indicators were evaluated. The authors compared the heat damage of raw milk with composite milk, obtained from raw milk and reconstituted milk (1 : 3), where the reconstituted milk was prepared from powdered milk (13.2%). Raw milk and composite milk were both processed from 65°C to 115°C for 15 sec, and lactulose, furosine, *β*-lactoglobulin, and lactoperoxidase were used as heat damage indices. The results showed that the lactulose content increased with heat treatment up to 95°C, and it was higher in composite milk due to the high amount of lactulose present in the original powdered milk. The furosine content considerably increased when temperature reached 75°C, but between 75 and 95°C it increased slowly. High temperatures (115°C) resulted in higher formation of lactulose than furosine, with a higher lactulose to furosine ratio in pasteurized milk than in composite pasteurized milk. The authors also found a better correlation between lactulose and furosine contents with the heat treatment of raw milk (*R*^2^ = 0.98) than in composite milk (*R*^2^ = 0.74). Therefore, the lactulose to furosine ratio may be a potential indicator for assessing raw milk overheating or reconstituted milk addition to raw milk heated at different temperatures (65°C to 115°C for 15 sec). Lactoperoxidase activity decreased with both heat treatment and reconstituted milk addition, and it was undetectable at 85°C. *β*-LG decreased in pasteurized milk and the addition of reconstituted milk reduced its content. The authors observed a second order correlation between *β*-LG and furosine in pasteurized milk (*R*^2^ = 0.90), while a still negative but linear correlation was observed in composite milk samples (*R*^2^ = 0.94). Therefore, the authors [60] concluded that lactoperoxidase is suitable for monitoring mild heat pasteurization, while lactulose, furosine, and *β*-LG may be potential indicators for assessing high heat pasteurization or raw milk.

Similarly, the work of Cho et al. [61] evaluated the effects of heat treatments and storage conditions on lactulose and furosine content in milk in order to identify them as potential indicators for heat damage of milk. At the same time, the lactulose to furosine ratio was evaluated for assessing adulteration of fresh milk. Several milk samples were evaluated under different conditions of storage and heating parameters (temperature and time). The results showed an increase in lactulose and furosine content with increasing temperature and heating time, which also occurred in HTST and UHT milk during storage at 30°C. The addition of reconstituted milk also induced an increase in lactulose and furosine contents, but the increase in furosine was much higher than lactulose, resulting in a decrease of the lactulose/furosine ratio. The authors concluded that lactulose and furosine, as is well known, are good quality indicators for heat-treated milk but the value of their ratio allows better identification of the addition of reconstituted milk.

Heat treatment indicators may be helpful in detecting frauds in the dairy sector as reported by Resmini et al. [62]. The authors reported that if the furosine contents exceed 8.6 mg/100 g in peroxidase-positive pasteurized milk, reconstituted powdered milk or high-temperature treated milk were certainly added. Adulteration of UHT or in-bottle sterilized milk with reconstituted powdered milk may also be detected by estimating the furosine to lactulose ratio. Galactosyl-*β*-pyranone (an advanced Maillard compound) is instead suitable for distinguishing directly and indirectly heated UHT milk.

## 8. Conclusions

In milk processing, heat treatments represent the common practice to inhibit the microbial growth and, in order to detect any fraud, the reference methods are used to evaluate the changes of different milk components (enzyme activities or whey proteins): however, in literature nonreference methods based on single thermal treatment indicators are well described; they employ various techniques such as HPLC, capillary electrophoresis, Diffuse Reflectance Infrared Fourier Transform Spectroscopy, or electrochemical biosensors.

However, analytical techniques based on single heat treatment indicator are not able to describe the whole system, especially because many of these parameters can change during storage. Nowadays chemometric approaches are more and more applied by researchers in order to summarize the results of the multiple heat treatment indicators: in this way they are determined according to the official methods but merged to obtain an overall description of the system.

As an alternative, nonreference methods based on spectroscopic techniques, such as UV-Vis and fluorescence, have been widely used to identify heat treatments changes in milk: these techniques are fast and, very often, do not require sample preparation.

Recent techniques such as size-exclusion chromatography with online electrospray mass spectrometry or stable isotope ratio mass spectrometry are also used to identify changes in heat-treated milk, but they require high cost and/or skilled personnel.

## Figures and Tables

**Table 1:** Limit of detection (LOD) and limit of quantification (LOQ) in some thermal treatment indicators according to nonreference methods.

Single molecules	Matrix	Sensitivity	References
Furosine	Milk	LOD 0.07 mg/L; LOQ 0.25 mg/L	[16]
Lactulose	Pasteurised, UHT, and sterilised milk	LOD 2.5 mg/L	[17]
Lactulose	Pasteurised, UHT, and sterilised milk	LOD 0.17 mg/100 mL	[18]
Lactulose	UHT milk	LOD 0.013 mg/mL; LOQ 0.028 mg/mL	[20]
Lactulose	Sterilised milk and lactose-reduced yoghurt	LOD 2.5 mg/L	[21]
LAL	Caseinates and fresh cheeses	LOD 50 ppm/protein; LOQ 152 ppm/protein	[29]
HMF	Industrial processed milk	LOD 0.2 *µ*mol/L	[35]
HMF	Milk	LOD 0.067 *μ*g/mL	[36]

LAL lysinoalanine; HMF 5-hydroxymethylfurfural; UHT ultrahigh temperature.
